# Research on Throughput-Guaranteed MAC Scheduling Policies in Wireless Networks

**DOI:** 10.3390/e24091246

**Published:** 2022-09-04

**Authors:** Fan Zhang, Baozhu Li, Gangqiang Yang

**Affiliations:** 1 School of Information Engineering, Shandong Management University, Jinan 250357, China; 2 Internet of Things & Smart City Innovation Platform, Zhuhai Fudan Innovation Institute, Zhuhai 519031, China; 3 School of Information Science and Engineering, Shandong University, Qingdao 226237, China

**Keywords:** MAC scheduling, throughput-guaranteed, end-to-end average delay

## Abstract

In wireless networks, MAC scheduling methods can be divided into two types according to the implementation model: centralized and distributed scheduling. By reasonably designing MAC scheduling policies, both centralized and distributed schedulers can ensure a reliable throughput capacity region, i.e., realizing throughput-guaranteed. However, it can be found that some existing throughput-guaranteed scheduling schemes cannot further ensure bounded end-to-end average delay, and the reason for this phenomenon has not been deeply analyzed. In practical communication networks, throughput and delay are equally important. Based on this idea, the existing MAC scheduling strategies are investigated systematically in this paper from two aspects of throughput and delay, and their performances are evaluated and compared through both theoretical analysis and simulation experiments. The work of this paper provides a theoretical basis for the improvement of MAC scheduling technology in the next-generation wireless networks.

## 1. Introduction

Depending on the way of obtaining the permission to use wireless channels, MAC layer protocols for wireless networks can be divided into two categories: competitive protocol and scheduling policy. The characteristic of the competitive protocol is that the node participates in the channel competition to win the permission to use the wireless channel. CSMA/CA protocol in IEEE 802.11 standard is a typical competitive protocol. Differently, the access permission of the wireless channel under the scheduling policy is pre-assigned in order to avoid the competition and conflict in transmission. The essence of MAC scheduling technology is to use STDMA (Space Time Division Multiple Access) to determine the scheduled time-slot for each link and allocate conflicting links into different time-slots [1]. When the network load is heavy or users have high requirements for bandwidth and delay, competitive protocols compete for resources relying on random access, which causes network congestion inevitably. By contrast, the scheduling policy allocates resources more reasonably based on STDMA. On one hand, MAC scheduling can provide higher bandwidth utilization because the link transmission time-slot is determined in advance. On the other hand, a well-designed MAC scheduling strategy can not only ensure reliable throughput performance but also reduce the data transmission delay, which is necessary for services that are sensitive to transmission bandwidth and delay (such as multimedia video) [2]. Therefore, MAC scheduling policy would become the first choice of MAC layer protocol in the next-generation wireless network systems.

Currently, widely used wireless networks can be classified into two types: centralized and distributed networks. A centralized network requires a central node for network control and management, such as CN (cellular networks). In distributed architecture, network nodes complete resource allocation by themselves, such as MANET (mobile ad hoc networks), WSN (wireless sensor networks) and WMN (wireless mesh networks). In order to adapt to different network architectures, a series of centralized and distributed MAC scheduling strategies have been proposed. One of the important criteria for evaluating the performance of MAC scheduling is the strong or weak stability of network systems. Reasonable MAC scheduler can maintain the strong or weak stability of the network system. When the input load locates within a certain capacity region, the system is defined as weakly stable [3] if the network can bear the effective throughput of all data packets. A scheduling policy is considered to be throughput-guaranteed as long as it keeps the network weakly stable under some interference model. Furthermore, the network system remains strongly stable if the end-to-end transmission delay can be upper bounded on the basis of ensuring weak stability [4]. Studies show that strong stability can significantly reduce the transmission delay of business flows from source to destination compared with weak stability, which improves the throughput efficiency and user experience [5]. At present, a series of research achievements have been made on designing MAC scheduling strategies to ensure strong/weak stability of wireless networks, which are elaborated in the Section 2.

**Figure 1:**
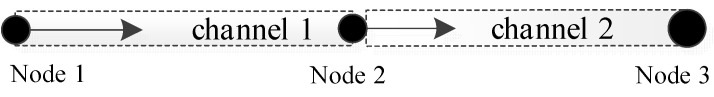
Multi-channel data transmission.

In addition, some researchers aim at extending MAC scheduling methods from single-channel networks to multi-channel networks. In multi-channel environment, the spectrum resource is divided into multiple non-overlapping channels, and the wireless interface (network card) of the node can choose one channel to complete communication. In this way, links in the same area are able to utilize different channels to transmit data simultaneously without interference. Hence, conflicts are avoided and the bandwidth is increased [6]. When a wireless node is configured with multiple interfaces, it can send and receive message at the same time-slot, which further improves the resource utilization [7]. The multi-channel data transmission is shown in Figure 1. In the physical layer, MIMO (Multiple Input and Multiple Output) and CR (Cognitive Radio) provide technical support for use of multiple channels. However, the application of multi-channel technology brings more complicated problems from hardware and software implementation. Due to channel difference and interface conflicts, it seems to be more difficult to design throughput-guaranteed MAC scheduling strategies for multi-channel wireless networks than for single-channel networks [8]. A series of MAC scheduling methods ensuring QoS (Quality of Service) have been proposed for multi-channel networks, which will be elaborated in the Section 2.

This paper focuses on MAC scheduling policies in single-channel and multi-channel wireless networks, proposes a complete MAC scheduling performance evaluation system, and uses this system to evaluate the throughput and delay performance of the existing typical wireless MAC scheduling policies. The rest of this paper is organized as follows. Section 2 introduces the existing centralized and distributed MAC scheduling strategies in both single-channel and multi-channel scenarios. Section 3 gives the judgment theorems of weak stability and strong stability of the networks, which constitute the performance evaluation system of MAC scheduling strategies. In Section 4, the throughput and delay performances of the existing MAC scheduling methods are analyzed. Section 5 completes the computer simulation experiments to verify the theoretical analysis. Finally, we conclude in Section 6. To improve readability, the acronyms used in the paper are listed in Table 1.

## 2. Existing MAC Scheduling Policies

This section comprehensively investigates and analyzes the existing typical MAC scheduling strategies proposed by researchers in recent years from three aspects: centralized scheduling under single-channel networks, distributed scheduling under single-channel networks, and scheduling for multi-channel networks.

### 2.1. Centralized Scheduling for Single-Channel Networks

A centralized MAC scheduling policy applies to the scenario with a control center. The central node (or base station) allocates the resources. Aiming at designing throughput-guaranteed centralized scheduling algorithms in single-channel networks, Tassiulas and Ephremides proposed a single-path-based Maximum Weighted Scheduler (MWS) [9] and a Back-Pressure Scheduler (BPS) for multipath environments [10]. Both MWS and BPS are proven to achieve the maximum throughput capacity region, which is the union of capacity regions realized by all possible scheduling mechanisms. In order to reduce the implementation complexity of MWS, Lin et al. proposed a Greedy Maximal Matching (GMM) algorithm that can realize centralized scheduling with much lower complexity than the MWS [11]. Ghiasian and Kar et al. described the delay performance of MWS based on the coloring number in graph theory and pointed out that MWS can guarantee the tightness of the upper bound of average delay only when the network topology is regular graphics [12,13]. Zhang et al. developed a Delay-enhanced Maximum Weight Scheduler (DMWS) based on specific load factors and proved that a DMWS can achieve the tightness of the upper bound of average delay under any network topology [14]. In addition, Alper et al. used a claw-free conflict graph to reduce the implementation complexity of the MWS algorithm and ensure transmission delay [15]. It can be seen from the above research results that both the throughput and delay performances of the centralized scheduling strategy for single-channel networks have been analyzed and improved.

### 2.2. Distributed Scheduling for Single-Channel Networks

For network architectures such as WSN, WMN and MANET where there is no central control node in the network, using the distributed MAC scheduling strategy is obviously more suitable than the centralized scheduling policy. In single-channel environments, Wu and Joo et al. considered the different interference models and proposed the distributed Maximal Scheduler (MS) [16,17]. It was proven that MS can achieve a reliable throughput capacity region with much lower implementation complexity than that of the centralized MWS algorithm. In order to further reduce the complexity of the distributed scheduler, Gupta and Zhang et al. proposed a Queue-Length Scheduler (Q-SCHED) based on random access technology under which each node sends data selectively according to a pre-calculated probability [18,19]. Q-SCHED can guarantee a throughput capacity region arbitrarily close to MS, and its implementation complexity does not increase with the number of network links. Based on this feature, Q-SCHED is more suitable for deployment in large-scale intensive wireless distributed networks. Furthermore, Bermond et al. put forward a throughput-guaranteed cross-layer scheme that combines Q-SCHED in the MAC layer and a path selection mechanism in the network layer for a multi-hop multi-path environment [20]. Both MS and Q-SCHED can maintain the weak stability of the network when the input load locates inside the capacity region. However, it has not been analyzed and verified whether the distributed MS and Q-SCHED can provide bounded end-to-end delay.

### 2.3. Scheduling for Multi-Channel Networks

Because of the utilization of multiple non-overlapping orthogonal channels in a multi-channel network environment, the channel and interface allocation need to be considered, which brings a technical challenge to the design of an efficient MAC scheduling strategy. For centralized scheduling, Lin and Choi et al. proved that both MWS and GMM used in single-channel networks can be extended straightforward to multi-channel networks without performance loss [21,22]. However, for distributed MAC scheduling, the direct extension of the single-channel scheduler to the multi-channel environment may lead to an extremely poor throughput performance due to the existence of channel differences [23]. Therefore, Lin and Zhang et al. designed a distributed Single-Path (SP) scheme for a single-path data flow scenario and its extension version Multi-Path (MP) policy for flows with multiple paths [24,25]. SP and MP utilize the idea of relay forwarding to complete data allocation and channel assignment simultaneously in the scheduling phase. Bhandari et al. developed a strategy without relay forwarding in multi-channel single-interface networks where each node is equipped with only one network card [26]. Cheng et al. proposed an improved mathematical model by using tuples. Based on this model, the single-channel MS strategy can be directly extended to multi-channel networks without a loss of throughput performance [27]. Zhang et al. developed a Distributed Algorithm with Low Complexity (DA-LC) for a multi-channel multi-interface environment by using the idea of single-channel Q-SCHED policy [28]. Moreover, a Low-Complexity distributed Channel assignment and Scheduling (LDCS) policy using packet information exchange technology in multi-channel single-interface WSN was proposed in [29]. The implementation complexity of DA-LC or LDCS does not increase with the network size. Through analysis, one can obtain that the above-mentioned distributed strategies for multi-channel networks are throughput-guaranteed. However, the delay performance is still unknown and needs further analysis.

### 2.4. Main Contribution

As we can see, designing a QoS-guaranteed MAC scheduling technology for wireless networks has attracted extensive attention, and a well-designed MAC scheduler has become an important technical means for solving the problem of resource allocation in wireless networks. In both single-channel and multi-channel networks, the existing centralized scheduling strategies are considered to achieve strong stability of the networks. As to distributed scheduling, the above-mentioned algorithms including MS, Q-SCHED, SP, MP, Tuple-based MS, DA-LC and LDCS can ensure the weak stability of the network when the input load locates within the throughput capacity region. However, whether these distributed policies can further ensure strong stability of the networks, relevant studies have not reached corresponding conclusions. In this paper, a complete MAC scheduling policy performance evaluation system is proposed, which consists of the principles and judgment theorems of network stability. Based on the evaluation system, both throughput and end-to-end delay performances of MAC scheduling policies in single-channel and multi-channel networks can be explored in depth. Thus, the performances of the existing typical scheduling strategies are comprehensively investigated in this paper from two aspects of theoretical analysis and simulation experiments. The simulation experiments validate our theoretical analysis. The work of this paper provides strong theoretical support and experimental data for the subsequent improvement and application of MAC scheduling technology in the next-generation networks.

## 3. Performance Evaluation Mechanism of MAC Schedulers

This section introduces the queue update and stability models of wireless networks. Furthermore, the judgment theorems of network stability are presented that provide the theoretical basis for analyzing the throughput and delay performances of MAC scheduling strategies.

### 3.1. Queue Update Model

**Figure 2:**
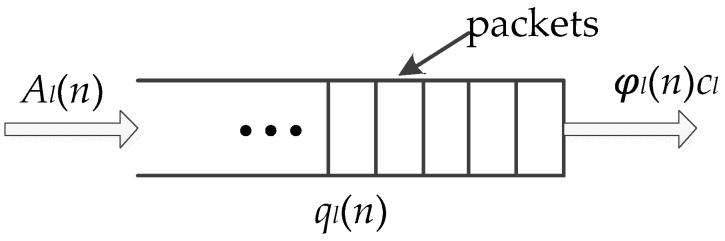
Queue Update Process in Wireless Networks.

Packets sent from the source node must be queued and forwarded to the destination node. The MAC scheduling method divides the time axis into time-slots and completes the input and output of the queue in each time-slot. Nodes are required to maintain data queues in both single-channel and multi-channel networks. The queue update action includes the arriving and sending of packets. According to the scheduling algorithm, when a queue is scheduled in this time-slot, data will leave the queue and be sent to the destination or to the next-hop node. Let *A_l_*(*n*) represent the number of packets arriving at link *l* at time-slot *n* and the value of *A_l_*(*n*) in each time-slot constitute a stochastic arrival process {*A_l_*(*n*)}. We usually assume that the random variables *A_l_*(*n*) in different time-slots are independent and identically distributed (i.i.d.) with a mean of *λ_l_*. It is further assumed that secondary moment is bounded, that is, we have Cov(*A_l_*(*n*), *A_k_*(*n*)) < ∞ for any two links *l* and *k*. Hence, the average input load of the system is ***λ*** = [*λ*_1_, …, *λ_L_*]. Let *q_l_*(*n*) denote the queue length of link *l* in time-slot *n*. Assume that the capacity of link *l* is fixed and denoted by *c_l_*. Indicator variable 𝜑*_l_*(*n*) is used to indicate whether link *l* is scheduled in time-slot *n*. If *l* is scheduled and *q_l_*(*n*) > 0, 𝜑*_l_*(*n*) = 1; otherwise, 𝜑*_l_*(*n*) = 0. Thus, the queue update process of any link *l* is shown in Figure 2.

The queue update process can be expressed as

(1) $$q_{l}(n+1)={\left[q_{l}(n)+A_{l}(n)-\varphi_{l}(n)c_{l}\right]}^{+}$$

where []^+^ is the projection on [0,∞). It can be seen from Equation (1) that the infinite discrete state stochastic process ***q***(*n*) = {*q_l_*(*n*), *l* = 1, …, *L*} constituted by all queue lengths is an irreducible and non-periodic Discrete Time Markov Chain (DTMC).

### 3.2. Stability Model

For wireless networks, weak/strong stability is an important basis for evaluating network performance, which directly determines the throughput and end-to-end delay of data flows. The following two theorems give definitions of weak stability and strong stability, respectively.

**Theorem** **1.** *For any υ > 0, if there exists a constant* Ψ>0*such that*

(2) $$\underset{n\to \infty }{\lim}\mathrm{Pr}\left\{\sqrt{\sum_{l=1}^{L}{\left[q_{l}(n)\right]}^{2}}>\Psi \right\}<\upsilon$$

*holds, the whole system is considered as weakly stable [4] where Pr{S} represents the probability of event S.*

Theorem 1 is actually Definition 2 in reference [4], which implies that weak stability can maintain the queue lengths finite. That is, the network system can bear the current input load and guarantee 100% throughput under the condition of weak stability. However, weak stability cannot further provide bounded end-to-end delay. In order to analyze the delay performance, the following theorem is introduced.

**Theorem** **2.** *For wireless networks, if the queue lengths satisfy*

(3) $$\underset{n\to \infty }{\lim\sup}\frac{1}{n}\sum_{\sigma =1}^{n}\sum_{l=1}^{L}E\left[q_{l}(\sigma )\right]<\infty ,$$

*then the network is considered to be strongly stable where E[] denotes the expectation.*

Theorem 2 is actually Definition 3 in reference [4]. If strong stability is satisfied, we have according to Little’s Law [30]:

(4) $$\bar{D}=\frac{\underset{n\to \infty }{\lim\sup}\frac{1}{n}\sum_{\sigma =1}^{n}\sum_{l=1}^{L}E\left[q_{l}(\sigma )\right]}{\sum_{l=1}^{L}\lambda_{l}}<\infty .$$

where $\bar{D}$ denotes the end-to-end average delay of all data flows. Hence, strong stability can guarantee an upper bound of the end-to-end average delay of data flows and can provide better network QoS than the weak stability state.

### 3.3. Judgment Theorems of Network Stability

Lyapunov analysis method [31] is always used to analyze the performance of a network queue system. A Lyapunov function is required to be constructed, which is defined as a nonnegative scalar measure function containing the information of all queue lengths in the network. Network performance is evaluated according to how much it affects the Lyapunov function between time-slots. For example, we can define the following Lyapunov function *V*(*n*) as

(5) $$V(n)=\sum_{l=1}^{L}c_{l}{\left[q_{l}(n)\right]}^{2}.$$

In network queue analysis, it is usually assumed that the queue length at the initial time is finite, that is, *E*[*q_l_*(0)] < ∞, for any *l* ∈ {1, ..., *L*}.

#### 3.3.1. Judgment Theorem of Weak Stability

After the Lyapunov function is constructed, Foster’s Criterion is needed to determine the weak stability of the system [32]. The criterion is applied to Markov queues with infinite nonperiodic countable states and provides a method for judging the positive recurrence and ergodicity of the Markov chains. The criterion is described as follows.

**Criterion** **1.** *For positive integer H and constant* ϵ, *if there exists a nonnegative Lyapunov function V(n) > 0 and a set* *Γ_0_ with finite states such that*

(6) $$E[V(n+H)-V(n)\left|q(n)\right.]<\infty \;\mathrm{if}\;q(n)\in \Gamma_{0}\;E[V(n+H)-V(n)\left|q(n)\right.]<-\epsilon \;\mathrm{if}\;q(n)\notin \Gamma_{0}$$

*holds for any time-slot n, then the aperiodic countable Markov chain (n) is positive recurrent and ergodic where E[V(n + H)* − ***q** V(n) │(n)] is defined as Lyapunov drift.*

According to Theorem 1, weak stability can be attained if the Markov chain ***q***(*n*) is positive recurrent [4]. Therefore, Foster’s criterion is the standard criterion for judging the weak stability of the network system.

#### 3.3.2. Judgment Theorem of Strong Stability

The Judgment of strong stability is not premised on Foster’s criterion, which is described as follows.

**Criterion** **2.** *If there exists a positive integer H > 0 such that E{**q**(τ)} < ∞ holds for τ* *∈ {0,* *⋯, H−1} and simultaneously for any time-slot n,*

(7) $$E[V(n+H)-V(n)\left|q(n)\right.]\leq B-\epsilon \sum_{l=1}^{L}q_{l}(n)$$

*holds for some B > 0,* ϵ*> 0, then the network system is strongly stable and the upper bound of the sum of average queue lengths is given by*

(8) $$\underset{n\to \infty }{\lim\sup}\frac{1}{n}\sum_{\tau =0}^{n-1}\sum_{l=1}^{L}E[q_{l}(\tau )]\leq \frac{B}{\epsilon }.\;$$

For the proof of Criterion 2, one can refer to [33]. According to Theorem 2 and Little’s Law, inequality (8) leads to an upper-bounded end-to-end average delay. If (7) is satisfied, then for any *δ* > 0 we have $\sum_{l=1}^{L}q_{l}(n)\geq \left(B+\delta \right)/\epsilon$ and $E[V(n+H)-V(n)\left|q(n)\right.]\leq -\delta$ hold. In other words, Criterion 2 can guarantee that the Lyapunov drift is negative when the queue lengths in the system are extremely large. Define a bounded state space as

(9) $$\Gamma_{0}=\left\{q(n)\geq 0\left|\sum_{l=1}^{L}q_{l}(n)\leq \left(B+\delta \right)/\epsilon \right.\right\}.$$

It can be seen that if ***q***(*n*) ∈ Γ_0_, we have *E*[*V*(*n* + *H*)−*V*(*n*)│***q***(*n*)] < ∞; otherwise, if ***q***(*n*) ∉ Γ_0_, *E*[*V*(*n* + *H*)−*V*(*n*)│***q***(*n*)] ≤−*δ*. Based on Criterion 1, weak stability can be deduced. Therefore, strong stability can lead to weak stability, not vice versa.

In this paper, we use Ω to represent the maximum throughput capacity region that can be ensured by any scheduling strategy. Under a particular MAC scheduling mechanism, if the network system remains weakly stable when the input load ***λ*** ∈ *γ* Ω (0 < *γ* < 1), the scheduling policy is then considered to be throughput-guaranteed and its efficiency ratio is *γ*. In fact, the efficiency ratio *γ* determines the throughput performance of a MAC scheduling policy.

## 4. Throughput and Delay Analysis of Typical MAC Scheduling Policies

This section uses Criterion 1 and Criterion 2 to evaluate the throughput and delay performances of several typical scheduling policies.

### 4.1. Performance Analysis of Scheduling Policies for Single-Channel Networks

For centralized scheduling in single-channel networks, the performance of the MWS [9] algorithm is analyzed. For distributed scheduling, we evaluate the performances of the MS [16] and the Q-SCHED strategies [18], respectively.

#### 4.1.1. Performance Evaluation of MWS

*Algorithm implementation*: In each time-slot *n*, the central control node in the network determines the schedule $\varphi_{\mathrm{MWS}}$ according to the following formula:

(10) $$\varphi_{\mathrm{MWS}}=\underset{\varphi \in S}{\arg\max}\sum_{l=1}^{L}q_{l}(n)c_{l}\varphi_{l}(n)$$

where *S* represents the set of all possible schedules under a particular interference model and the schedule vector ***φ*** = [*φ*_1_(*n*), …, *φ_L_*(*n*)]. Obviously, the realization of MWS requires a central node to execute the scheduling process on the premise of knowing all the queue length information in the network and to determine the schedule in each time-slot through Equation (10). Hence, the implementation complexity of MWS is high.

*Performance evaluation of MWS*: Assume that the input load vector ***λ*** locates strictly inside the maximum capacity region. That is, there exists an L-dimensional vector ***ε*** with all elements equal to *ε* such that ***λ*** + ***ε*** ∈ Ω holds. For time-slot *n*, the following Lyapunov function is constructed as

(11) $$V(n)=\sum_{l=1}^{L}{\left[q_{l}(n)\right]}^{2}.$$

Then, one-step Lyapunov drift $\Delta (q(n))=E[V(n+1)-V(n)\left|q(n)\right.]$ can be upper bounded by

(12) $$\Delta (q(n))\leq E\left\{{\left[A_{l}(n)-\varphi_{l}(n)\right]}^{2}\right\}-2\varepsilon \sum_{l=1}^{L}E\left[q_{l}(n)\right]$$

Thus, one can refer to Theorem 1 in reference [34] and obtain that

(13) $$\underset{n\to \infty }{\lim\sup}\frac{1}{n}\sum_{\tau =0}^{n-1}\sum_{l=1}^{L}E[q_{l}(\tau )]\leq \frac{\underset{n\to \infty }{\lim\sup}\frac{1}{n}\sum_{\tau =0}^{n-1}\sum_{l=1}^{L}E\left\{{\left[A_{l}(\tau )-\varphi_{l}(\tau )\right]}^{2}\right\}}{2\varepsilon }<\infty .$$

Therefore, the network is strongly stable according to Theorem 2. Under the stability condition, we have $\lim\sup_{n\to \infty }\frac{1}{n}\sum_{\tau =0}^{n-1}\sum_{l=1}^{L}E[\varphi_{l}(\tau )]=\lambda_{l}$ and (13) can be rewritten as

(14) $$\underset{n\to \infty }{\lim\sup}\frac{1}{n}\sum_{\tau =0}^{n-1}\sum_{l=1}^{L}E[q_{l}(\tau )]\leq \frac{\sum_{l=1}^{L}\lambda_{l}+\sum_{l=1}^{L}E\left[A_{l}{\left(n\right)}^{2}\right]-2\sum_{l=1}^{L}\lambda_{l}{}^{2}}{2\varepsilon }.$$

Using Little’s Law, the upper bound of the average delay $\bar{D}$ is given by

(15) $$\bar{D}\leq \frac{\sum_{l=1}^{L}\lambda_{l}+\sum_{l=1}^{L}E\left[A_{l}{\left(n\right)}^{2}\right]-2\sum_{l=1}^{L}\lambda_{l}{}^{2}}{2\varepsilon \sum_{l=1}^{L}\lambda_{l}}.$$

It can be seen from the analysis above that MWS can achieve the maximum capacity region and can ensure the strong stability of the network when the input load locates inside the capacity region. The upper bound of average end-to-end delay is given by (15), which depends on the arrival rates and the parameter *ε*.

#### 4.1.2. Performance Evaluation of MS

*Algorithm implementation*: MS is a typical distributed scheduling strategy. Under MS, any queue that meets *q_l_*(*n*) > *c_l_* is scheduled in time-slot *n* unless another queue in its interference set *I*(*l*) has already been scheduled. Hence, employing MS can ensure

(16) $$\sum_{k\in I(l)}\frac{q_{k}(n)}{c_{k}}\geq 1.$$

holds for each link *l*. MS does not require the centralized control node and each link decides whether to participate in the data transmission at the current time-slot. The implementation complexity of MS is much lower than that of the MWS policy.

*Performance evaluation of MS*: Assume that $\sum_{k\in I(l)}\lambda_{k}<1$ holds for each link *l*, we use the following Lyapunov function:

(17) $$V(n)=\sum_{l=1}^{L}q_{l}(n)\left(\sum_{k\in I(l)}q_{k}(n)\right).$$

Then the one-step Lyapunov drift Δ(q(n)) satisfies

(18) $$\begin{array}{c} \Delta (q(n))=E[V(n+1)-V(n)\left|q(n)\right.]\\ \;\;=-2\omega \sum_{l=1}^{L}q_{l}(n)+B. \end{array}$$

where *ω* and *B* are positive numbers and $\omega =1-\underset{l}{\max}\sum_{k\in I(l)}\lambda_{k}$. Thus, according to Criterion 2, the entire network system is strongly stable, and the end-to-end average delay has an upper bound given by

(19) $$\bar{D}=\frac{\underset{n\to \infty }{\lim\sup}\frac{1}{n}\sum_{\tau =0}^{n-1}\sum_{l=1}^{L}E[q_{l}(\tau )]}{\sum_{l=1}^{L}\lambda_{l}}\leq \frac{B}{2\omega \sum_{l=1}^{L}\lambda_{l}}.$$

Satisfying $\sum_{k\in I(l)}\lambda_{k}<1$ implies that MS is throughput-guaranteed and the efficiency ratio of MS is 1/*K*, where *K* is the interference degree [17].

#### 4.1.3. Performance Evaluation of Q-SCHED

**Figure 3:**
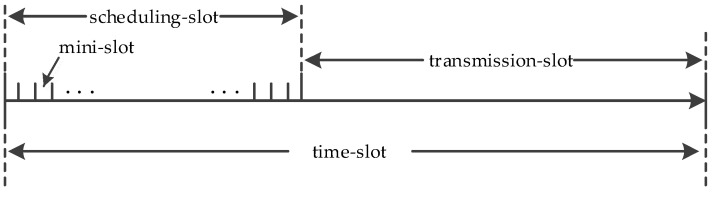
Time-slot Division under Random Access Mechanism.

*Algorithm implementation*: Different from MS, the distributed Q-SCHED strategy divides each time-slot into scheduling-slot and transmission-slot, and further divides the scheduling-slot into *M* mini-slots, as shown in Figure 3.

In each time-slot, link *l* picks the backoff time from the set {1, 2, …, *M* + 1} according to the following probability:

(20) $$\begin{array}{c} \mathrm{Pr}\left\{I=M+1\right\}=e^{-p_{l}}\\ \mathrm{Pr}\left\{I=m\right\}=e^{-p_{l}\frac{m-1}{M}}-e^{-p_{l}\frac{m}{M}}\;\;\;\;\;\;\;\;\;m=1,2,\ldots ,M. \end{array}$$

where *I* represents the backoff time selected by *l* and the parameter *p_l_* is calculated by

(21) $$p_{l}=\log M\cdot \frac{\frac{q_{l}(n)}{c_{l}}}{\max_{j\in I(l)}\left[\sum_{k\in I(j)}\frac{q_{k}(n)}{c_{k}}\right]}.$$

Picking *M* + 1 means that the link is not scheduled in this time-slot. Once the backoff time expires, the link starts transmitting unless there have been other links in its interference set that are sending data.

*Performance evaluation of Q-SCHED*: We introduce the following Lyapunov function:

(22) $$V(n)=\underset{l\in L}{\max}\left[\sum_{j\in I(l)}\frac{q_{j}(n)}{r_{j}}\right].$$

For some *μ* > 0, if the input load of the network satisfies

(23) $$\sum_{j\in I(l)}\frac{\lambda_{j}}{r_{j}}<1-\frac{\log M+1}{M}-4\mu$$

then there exists a positive constant Φ and a positive integer *H* such that [18]

$$E\left[V(n+H)-V(n)\left|q(n)\right.\right]<0$$

holds for *V*(*n*) ≥ Φ. Since {***q***(*n*): *V*(*n*) < Φ} is a set with finite states, we can obtain that the Markov chain {***q***(*n*)} is positive recurrent using Criterion 1. Hence, the network is weakly stable. It should be noted that ensuring weak stability under the condition (23) implies that the Q-SCHED policy is throughput-guaranteed. However, strong stability is not achieved, and Q-SCHED cannot provide a bounded end-to-end average delay. On the other hand, based on the random access technology as shown in Figure 3, the implementation complexity of Q-SCHED does not increase with the number of network links and nodes. In contrast, the MS strategy adopts the maximum matching mechanism that requires *O*(log*L*) number of iterations [18].

### 4.2. Performance Analysis of Scheduling Policies for Multi-Channel Networks

For scheduling in multi-channel networks, performances of the straightforward extension of MWS [22], distributed Tuple-based MS [27] and DA-LC [28] are evaluated, respectively.

#### 4.2.1. Performance Evaluation of the Straightforward Extension of MWS

*Algorithm implementation*: MWS in single-channel networks can be directly extended to multi-channel networks. Similar to MWS, the scheduling indicator vector $\varphi_{\mathrm{MWS}}^{*}$ is calculated by the central control node in each time-slot *n* according to

(24) $$\varphi_{\mathrm{MWS}}^{*}=\underset{\varphi \in S}{\arg\max}\sum_{l,c}q_{l}(n)r_{l}^{c}\varphi_{l}^{c}(n)$$

where $r_{l}^{c}$ indicates the transmission rate of link *l* operating on channel *c* and $\varphi_{l}^{c}(n)$ denotes the scheduling indicator variable by taking 1 when link *l* is scheduled on channel *c*, and 0 otherwise.

*Performance evaluation*: Literature [22] has proved that when the input load locates within the maximum capacity region, the positive recurrence of the Markov chain {***q***(*n*)} can be achieved. That is, the network system remains weakly stable when ***λ*** ∈ Ω. In fact, if we construct the following Lyapunov function

(25) $$V(n)=\sum_{l=1}^{L}{\left[q_{l}(n)\right]}^{2}$$

it is not difficult to prove by referring to [35] that MWS can also guarantee

(26) $$\underset{n\to \infty }{\lim\sup}\frac{1}{n}\sum_{\tau =0}^{n-1}\sum_{l=1}^{L}E[q_{l}(\tau )]<\infty$$

holds in multi-channel environment. Using Little’s Law, the network is strongly stable and can ensure a bounded end-to-end delay. 

#### 4.2.2. Performance Evaluation of Tuple-Based MS

**Figure 4:**
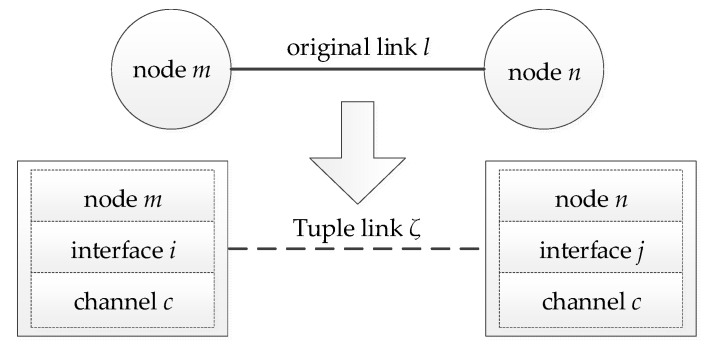
Transformation Process of Tuple-based Model.

*Algorithm implementation*: Authors of [27] proposed a mathematic model in which each node, channel, and interface are modeled as a Tuple. At the same time, the original links are transformed into Tuple links. Thus, the multi-channel environment can be virtualized into a single-channel network, as shown in Figure 4.

After the Tuple-based model is established, the MS algorithm is implemented based on Tuple links. Suppose *I*(*ζ*), *q_ζ_*(*n*) and *c_ζ_* represent the interference set, queue length and capacity of Tuple link *ζ* respectively. According to MS, we have

(27) $$\sum_{\gamma \in I(\zeta )}\frac{q_{\gamma }(n)}{c_{\gamma }}\geq 1.$$

holds for any Tuple link *ζ*.

*Performance evaluation*: To evaluate the performance of Tuple-based MS, the following Lyapunov function is constructed:

(28) $$V(n)=\sum_{\zeta }q_{\zeta }(n)\left(\sum_{\gamma \in I(\zeta )}q_{\gamma }(n)\right).$$

If $\sum_{\gamma \in I(\zeta )}\lambda_{\gamma }<1$ holds for each Tuple link *ζ*, one can achieve the one-step Lyapunov drift Δ(q(n)) as

(29) $$\begin{array}{c} \Delta (q(n))=E[V(n+1)-V(n)\left|q(n)\right.]\\ \;\;=-2\kappa \sum_{\zeta }q_{\zeta }(n)+B_{1}. \end{array}$$

where *κ* and *B*_1_ are positive numbers and $\kappa =1-\underset{\zeta }{\max}\sum_{\gamma \in I(\zeta )}\lambda_{\gamma }$. Thus, according to Criterion 2, the entire network system is strongly stable. Satisfying $\sum_{k\in I(l)}\lambda_{k}<1$ implies that the Tuple-based MS is throughput-guaranteed and the efficiency ratio of is 1/*Y*, where *Y* denotes the interference degree under the Tuple-based model.

#### 4.2.3. Performance Evaluation of DA-LC

DA-LC algorithm is a combination of the Tuple-based model and the Q-SCHED strategy in a single-channel network. Specifically, the multi-channel environment is first virtualized into a single-channel network using the transformation process shown in Figure 4. Then, the random-access-based Q-SCHED policy is implemented for the Tuple-based networks. To evaluate the performance of DA-LC, the following Lyapunov function is introduced:

(30) $$V(n)=\underset{\zeta }{\max}\left[\sum_{\gamma \in I(\zeta )}\frac{q_{\gamma }(n)}{r_{\gamma }}\right].$$

Then there exists a positive constant Φ and a positive integer *H* such that

(31) $$E\left[V(n+H)-V(n)\left|q(n)\right.\right]<0$$

holds for *V*(*n*) ≥ Φ. Hence, the network should be weakly stable by using Criterion 1 under DA-LC. The efficiency ratio of DA-LC can be arbitrarily close to that of Tuple-based MS. Therefore, compared with Tuple-based MS strategy, DA-LC cannot guarantee a bounded end-to-end average delay. However, the implementation complexity of DA-LC is much lower and does not increase with the number of Tuple links since it adopts the random access technology.

## 5. Simulation

**Figure 5:**
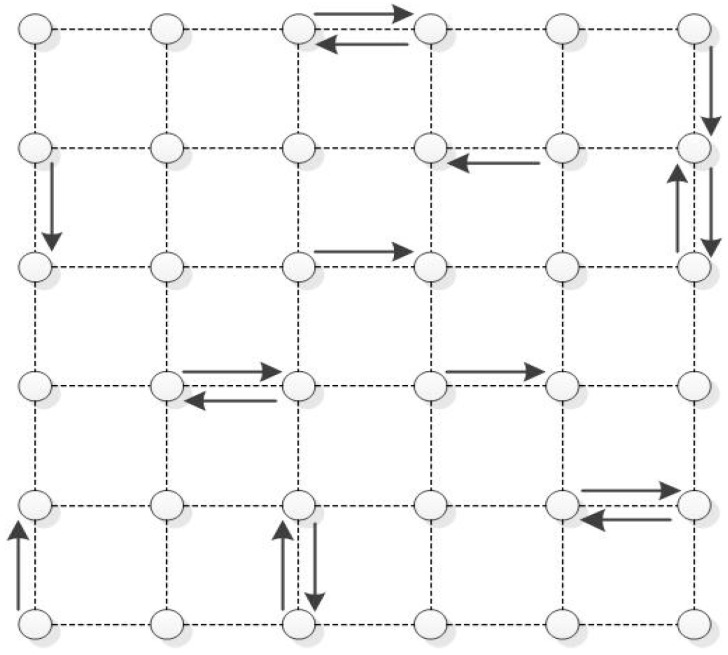
Network Topology for Experiments.

In this section, we use NS-2.31 software to simulate actual network scenarios and compare the throughput and delay performances of several existing wireless scheduling strategies. We use the topology shown in Figure 5, which has 36 nodes and 60 links. There are 16 data flows (represented by arrows) in the topology, and each data flow goes through a one-hop transmission. Assume that all data flows have the same data transmission rate (input load) of *λ*. In a single-channel environment, all links share one available channel to transmit data. In a multi-channel environment, the interface (network card) of a node can switch between four different channels with the channel capacity of 1, 1, 2, 2 respectively. The simulation parameters adopted in our experiments are shown in Table 2.

**Figure 6:**
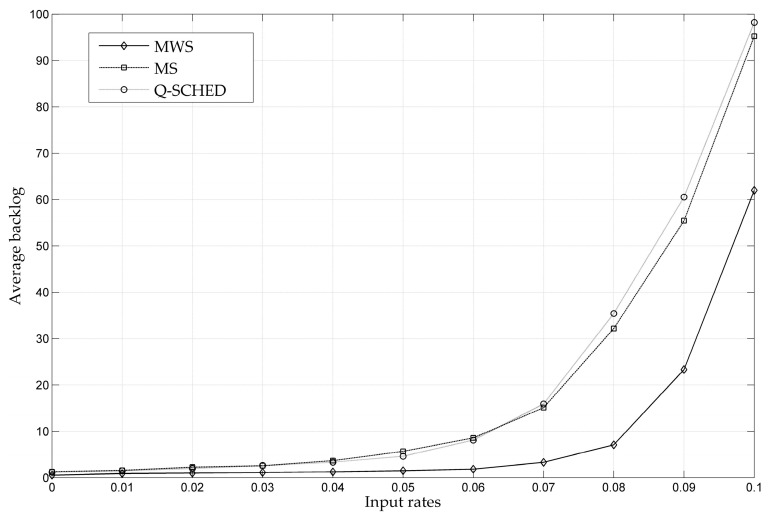
Throughput Performance Comparison of MWS, MS and Q-SCHED.

Figure 6 shows the throughput performance comparison of the centralized MWS, the distributed MS and the Q-SCHED strategies in single-channel environment. As we can see from the simulation data, the value of the average backlog increases sharply when the input load increases gradually to a certain threshold, which means that the input load has reached the boundary of the throughput capacity region that can be ensured by the scheduling policy. Compared with MS and Q-SCHED, MWS can guarantee a larger capacity region because MWS is a centralized throughput-optimal algorithm. At the same time, the throughput performances of MS and Q-SHCED are very close because the right-hand side of the inequality sign of (9) can arbitrarily approach to 1. In terms of implementation complexity, Q-SCHED has the lowest complexity due to the random access technology whereas the centralized implementation brings a high complexity to MWS.

**Figure 7:**
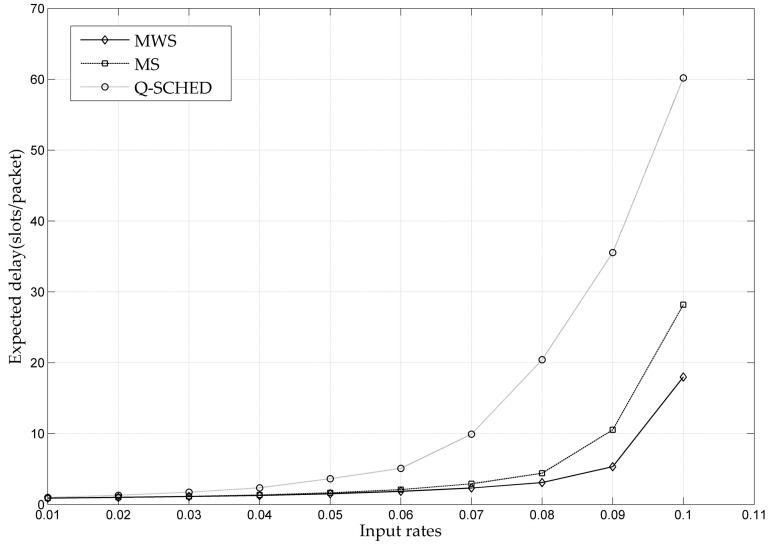
Delay Performance Comparison of MWS, MS and Q-SCHED.

Figure 7 gives the delay performance comparison of the MWS, MS and Q-SCHED algorithms. It can be seen from the figure that when the input load is small, the average end-to-end delay remains at a low level. When the input load increases further, the average delay increases significantly, indicating that congestion has occurred among the network queues. By comparison, it is found that MWS has the best delay performance, because it always selects the links with a larger queue length for sending data in each time-slot. The delay performance of Q-SCHED is the worst, and the average delay increases rapidly before the input load reaches the capacity boundary. The reason for this phenomenon is that Q-SCHED can only guarantee the weak stability of the network without ensuring strong stability, as analyzed in the previous section.

**Figure 8:**
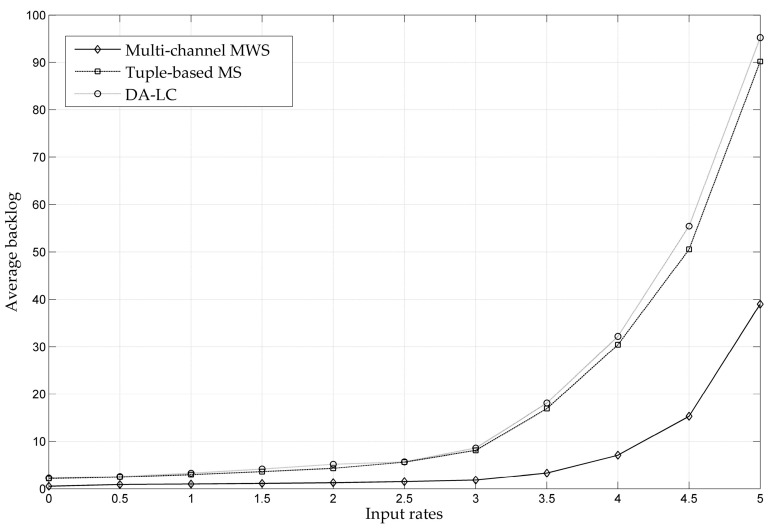
Throughput Performance Comparison of MWS, Tuple-based MS and DA-LC.

For a multi-channel network environment, Figure 8 shows the throughput performances of the multi-channel MWS, the Tuple-based MS and the DA-LC scheduling strategies. As we can see from the figure, the maximum throughput capacity region is significantly larger than that of the single-channel network due to the use of multiple channels. At the same time, the centralized MWS still has the best throughput performance of the three algorithms. The Tuple-based MS and the DA-LC both adopt the Tuple-based model and the distributed implementation method, and their throughput performances are close. In addition, the implementation complexity of the DA-LC is much lower than that of the MWS and Tuple-based MS.

**Figure 9:**
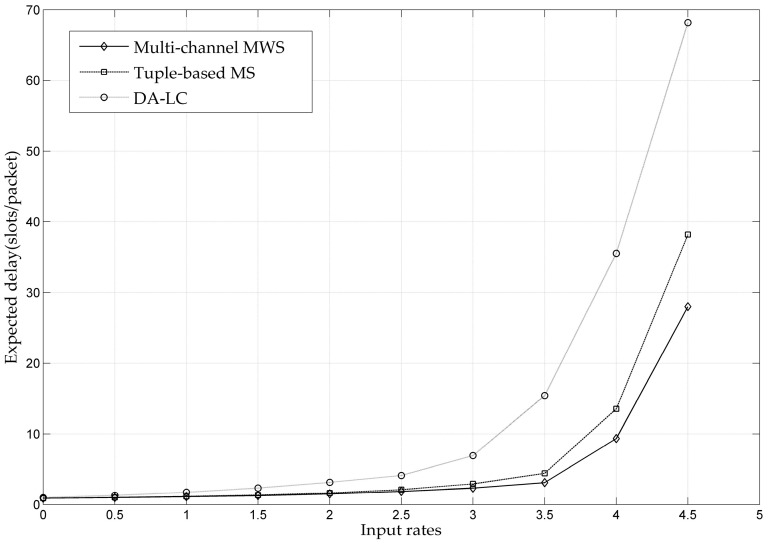
Throughput Performance Comparison of MWS, Tuple-based MS and DA-LC.

Figure 9 shows the end-to-end average delay performance of the MWS, Tuple-based MS and DA-LC algorithms. According to the analysis in the previous section, the MWS and Tuple-based MS can guarantee the strong stability of the network, whereas the DA-LC can only ensure the weak stability. Therefore, the delay performance of the MWS and Tuple-based MS is much better than that of the DA-LC, which can be clearly reflected in the figure. It can be obtained from the simulation experiments that the delay performance of the single-channel Q-SCHED or the multi-channel DA-LC scheduling policy is worse than that of other comparable scheduling algorithms. However, the implementation complexity of Q-SCHED or DA-LC does not increase with the network size. Hence, one can understand that they sacrifice delay for the reduction of complexity.

## 6. Conclusions

In the next-generation wireless networks, MAC scheduling is becoming the preferred technology of MAC layer. In order to better design the scheduling algorithms and verify their performances in different scenarios, this paper summarizes a complete set of methods, including the concepts of strong/weak stability and their judgment methods. By using these methods, this paper analyzes the performances of typical MAC scheduling policies, including throughput performance and delay performance. Through theoretical analysis and simulation experiments, we verify that strong stability can provide lower end-to-end delay than weak stability in both single-channel and multi-channel networks. The research work of this paper provides a theoretical basis for the design and evaluation of MAC scheduling policies.

## Figures and Tables

**Table 1:** List of acronyms used in this paper.

Acronym	Explanation
STDMA	Space Time Division Multiple Access
CN	cellular networks
MANET	mobile ad hoc networks
WSN	wireless sensor networks
WMN	wireless mesh networks
MIMO	Multiple Input and Multiple Output
CR	Cognitive Radio
MWS	Maximum Weighted Scheduler
BPS	Back-Pressure Scheduler
GMM	Greedy Maximal Matching
MS	Maximal Scheduler
Q-SCHED	Queue-Length Scheduler
SP	Single-path scheduler in multichannel networks
MP	Multi-path scheduler in multichannel networks
DA-LC	Distributed Algorithm with Low Complexity
Tuple-based MS	Tuple-based Maximal Scheduler
DTMC	Discrete Time Markov Chain

**Table 2:** Simulation parameters.

Parameter	Value
Software	NS-2.31
Simulation time	300 s
Number of nodes	36
Radio frequency	2.4 GHz
Number of links	60
Number of channels	4
Simulation terrain	500 m × 500 m
Path-loss model	Two-ray
Bandwidth	100 MHz
Number of flows	16
Data sessions	CBR
